# Supplementary figures and images for: Live imaging of the co-translational recruitment of XBP1 mRNA to the ER and its processing by diffuse, non-polarized IRE1α
Source: eLife. 2022 Jun 22;11:e75580. doi: 10.7554/eLife.75580 (PMC9217131; doi:10.7554/eLife.75580)

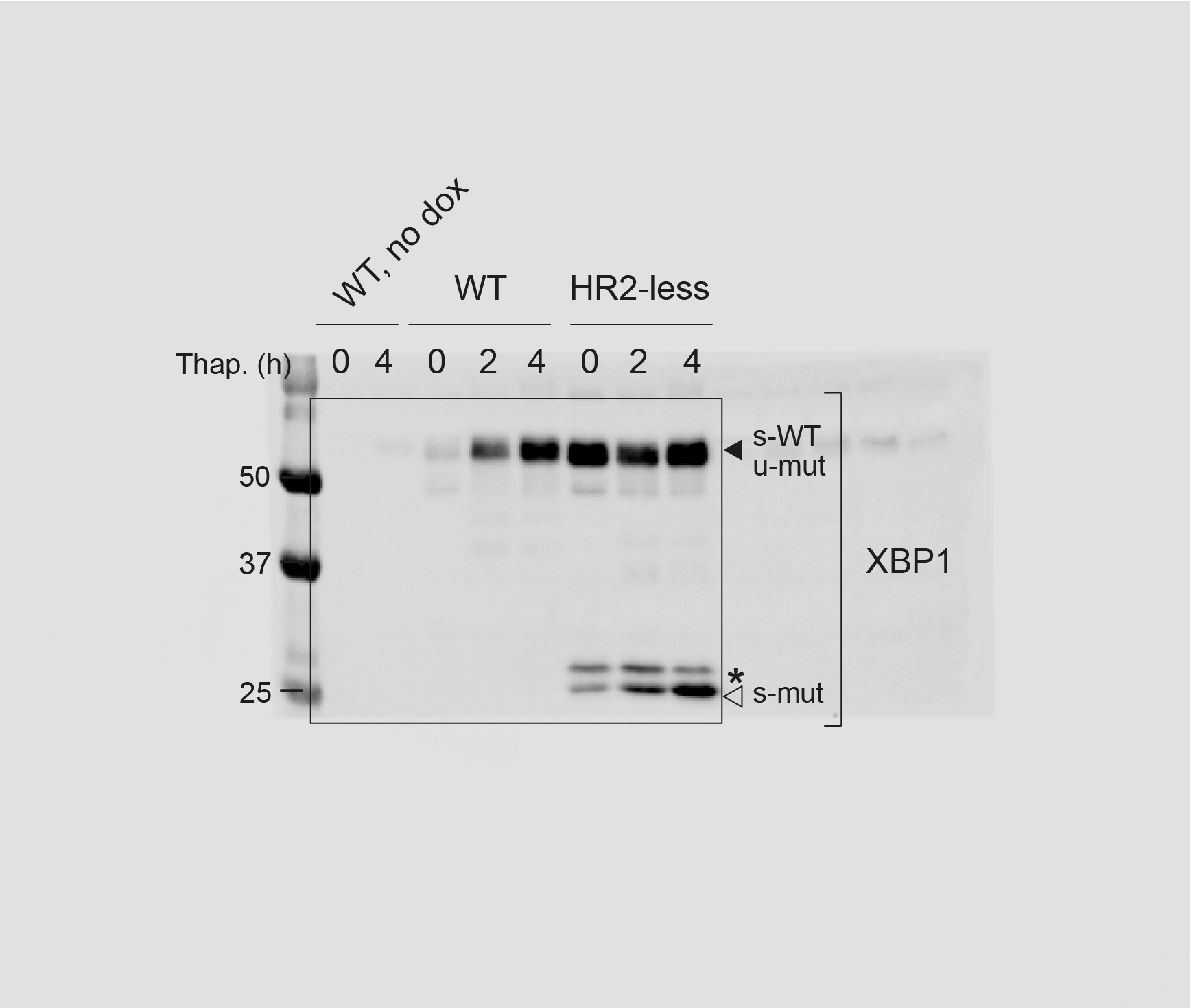

Supplement: Figure 1—source data 1. [file elife-75580-fig1-data1.zip › Figure 1-source data 1.tif]

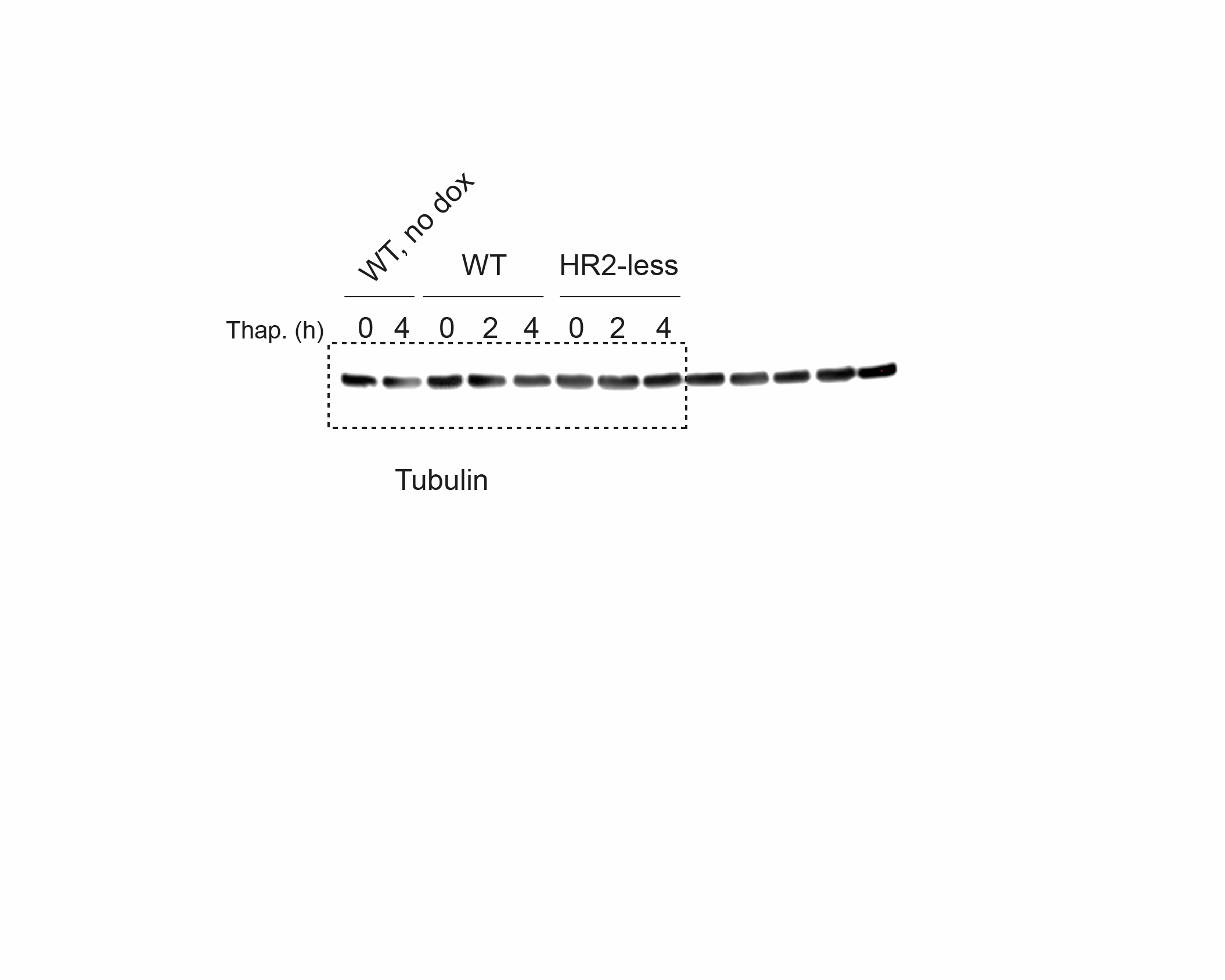

Supplement: Figure 1—source data 1. [file elife-75580-fig1-data1.zip › Figure 1-source data 2.png]

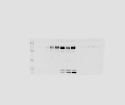

Supplement: Figure 1—source data 1. [file elife-75580-fig1-data1.zip › Figure 1-source data-raw images/0008099_01_TH.jpg]

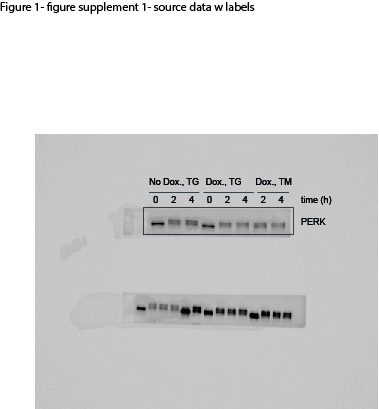

Supplement: Figure 1—figure supplement 1—source data 1. [file elife-75580-fig1-figsupp1-data1.zip › Figure 1- figure supplement 1- source data 1 w labels.jpg]

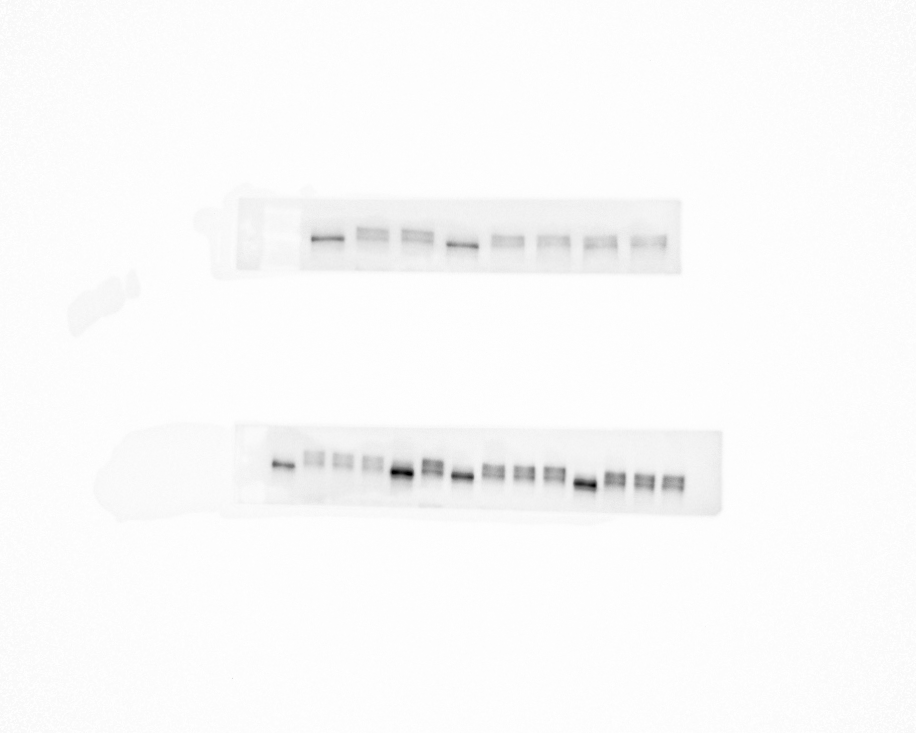

Supplement: Figure 1—figure supplement 1—source data 1. [file elife-75580-fig1-figsupp1-data1.zip › Figure 1- figure supplement 1- source data 1.raw16.tif]

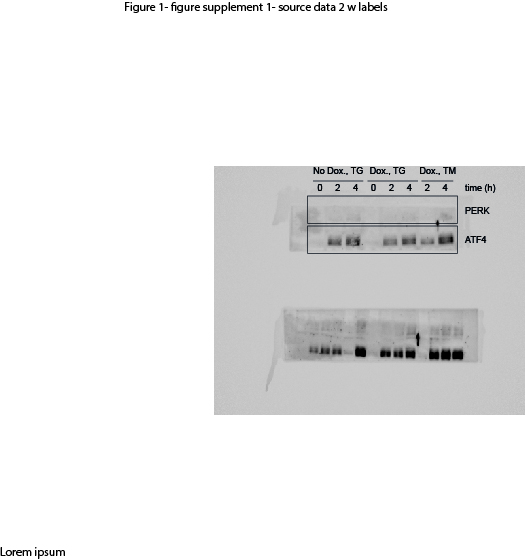

Supplement: Figure 1—figure supplement 1—source data 1. [file elife-75580-fig1-figsupp1-data1.zip › Figure 1- figure supplement 1- source data 2 w labels.jpg]

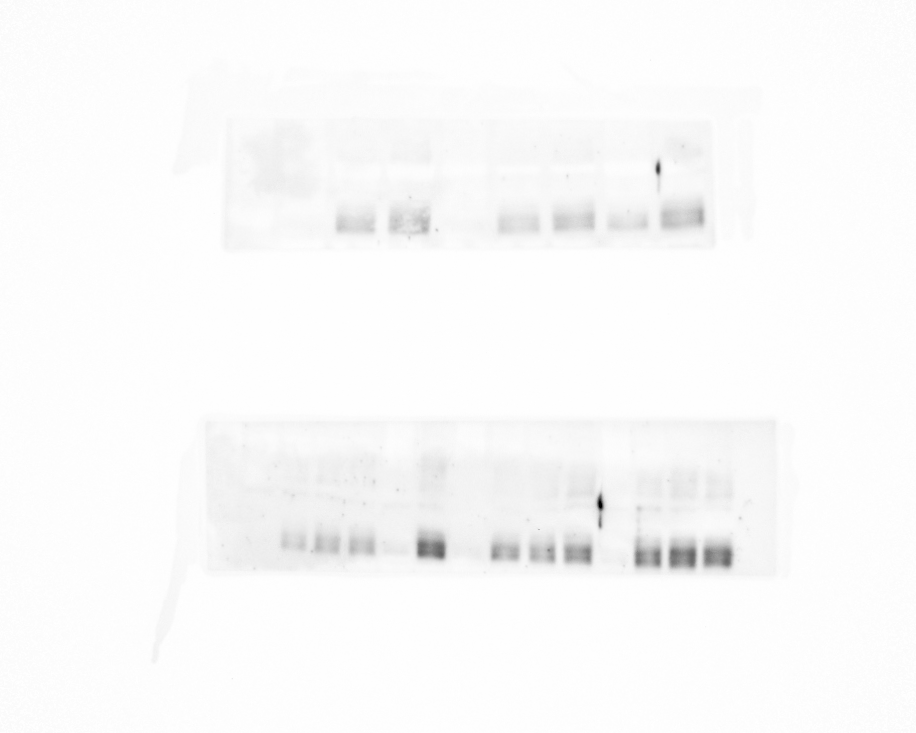

Supplement: Figure 1—figure supplement 1—source data 1. [file elife-75580-fig1-figsupp1-data1.zip › Figure 1- figure supplement 1- source data 2.raw16.tif]

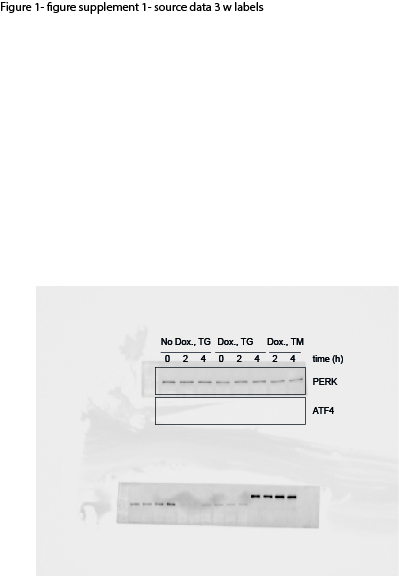

Supplement: Figure 1—figure supplement 1—source data 1. [file elife-75580-fig1-figsupp1-data1.zip › Figure 1- figure supplement 1- source data 3 w labels.jpg]

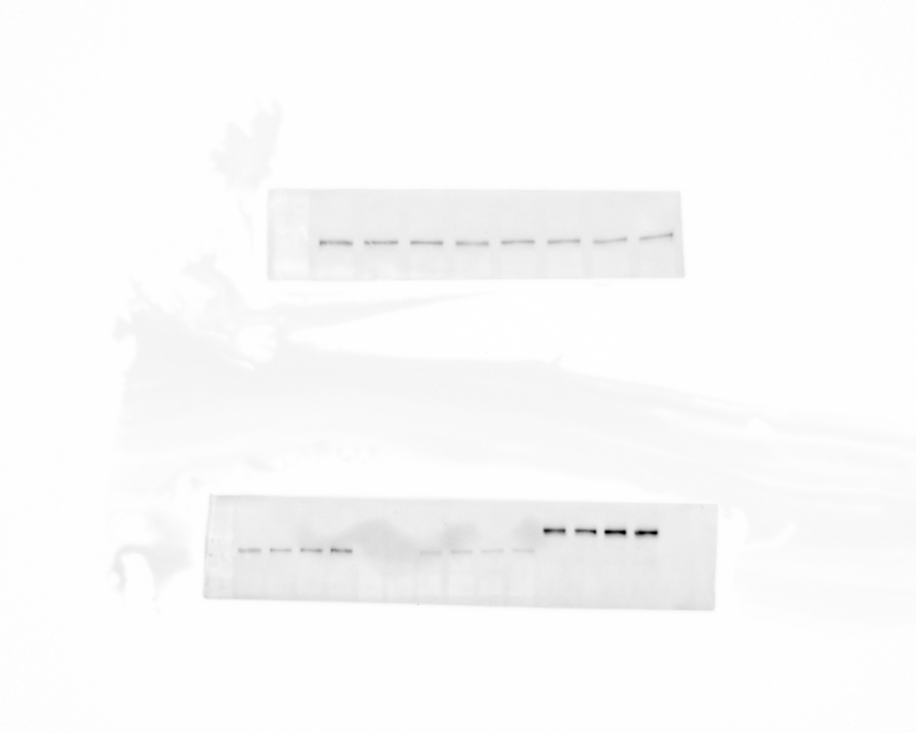

Supplement: Figure 1—figure supplement 1—source data 1. [file elife-75580-fig1-figsupp1-data1.zip › Figure 1- figure supplement 1- source data 3.raw16.tif]

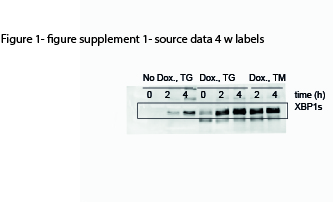

Supplement: Figure 1—figure supplement 1—source data 1. [file elife-75580-fig1-figsupp1-data1.zip › Figure 1- figure supplement 1- source data 4 w labels.jpg]

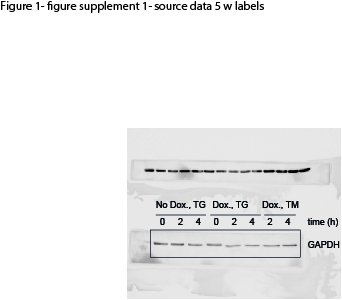

Supplement: Figure 1—figure supplement 1—source data 1. [file elife-75580-fig1-figsupp1-data1.zip › Figure 1- figure supplement 1- source data 5 w labels.jpg]

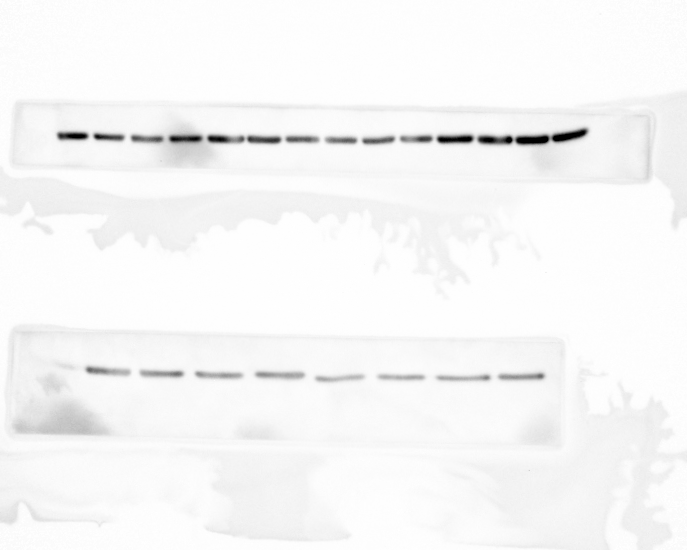

Supplement: Figure 1—figure supplement 1—source data 1. [file elife-75580-fig1-figsupp1-data1.zip › Figure 1- figure supplement 1- source data 5.raw16.tif]

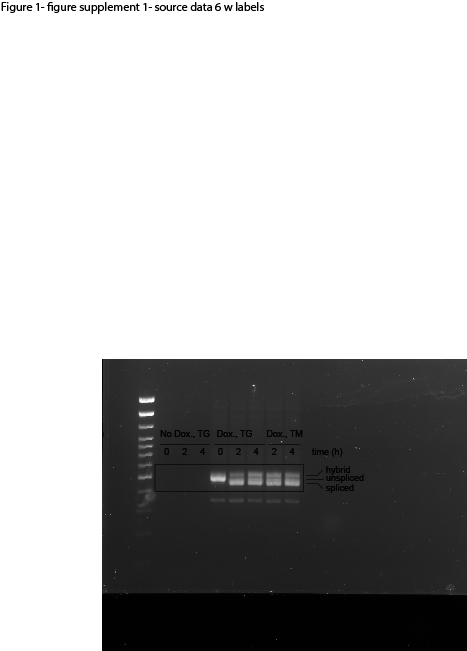

Supplement: Figure 1—figure supplement 1—source data 1. [file elife-75580-fig1-figsupp1-data1.zip › Figure 1- figure supplement 1- source data 6 w labels.jpg]

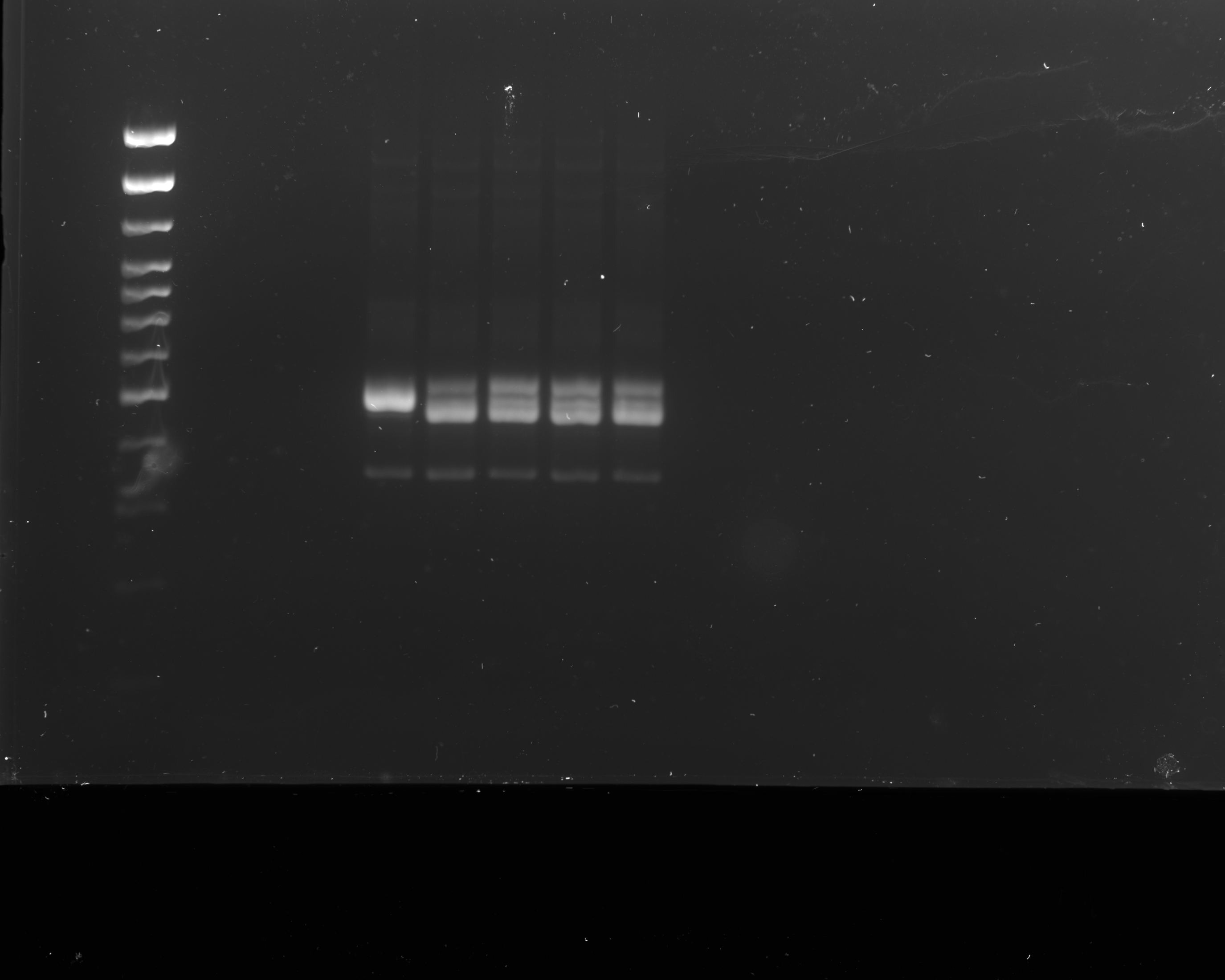

Supplement: Figure 1—figure supplement 1—source data 1. [file elife-75580-fig1-figsupp1-data1.zip › Figure 1- figure supplement 1- source data 6.tif]

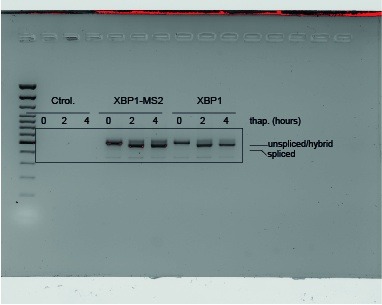

Supplement: Figure 1—figure supplement 1—source data 1. [file elife-75580-fig1-figsupp1-data1.zip › Figure 1- figure supplement 1- source data 7 w labels.jpg]

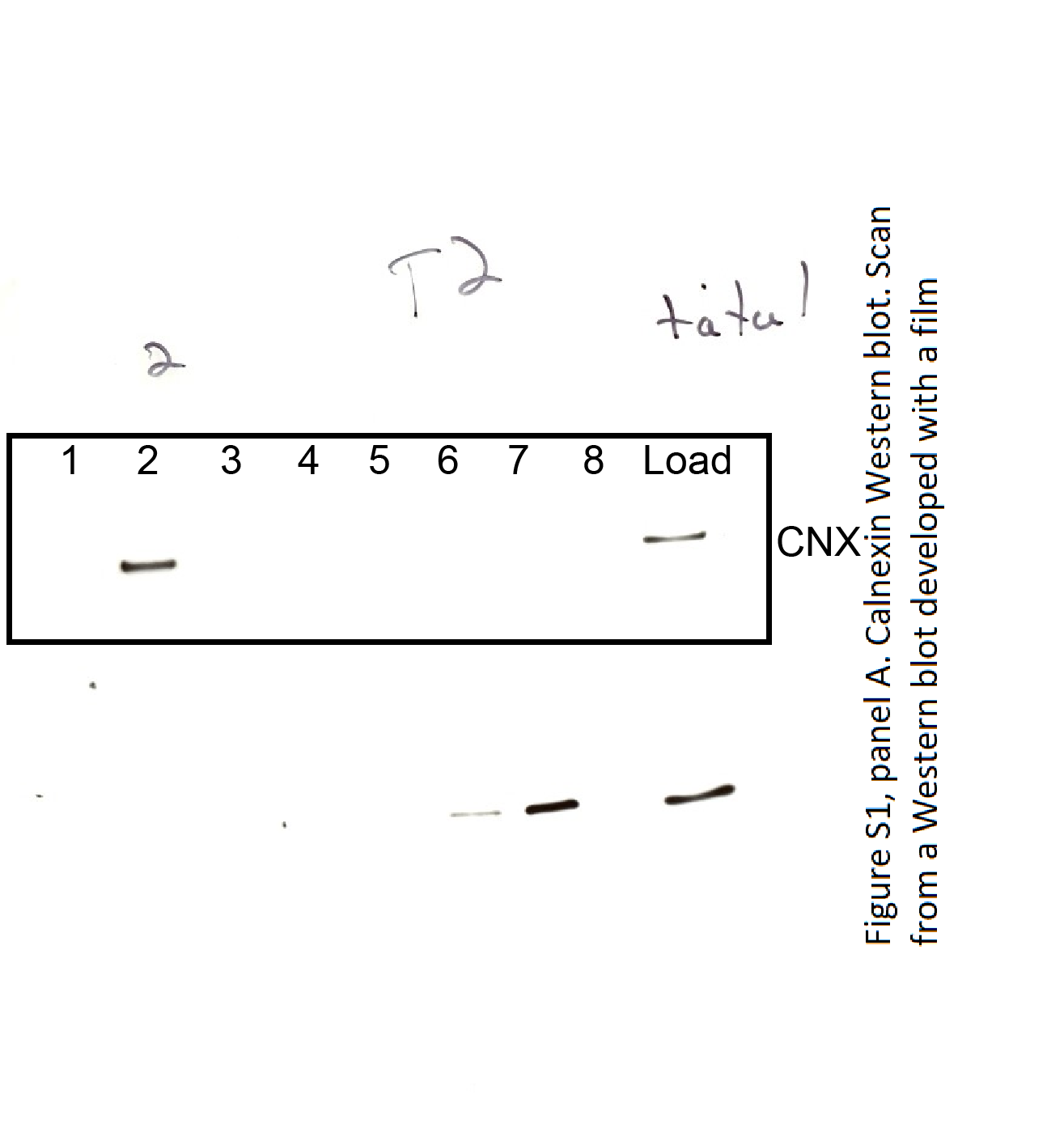

Supplement: Figure 1—figure supplement 2—source data 1. [file elife-75580-fig1-figsupp2-data1.zip › Figure 1-figure supplement 2 -source data 1.png]

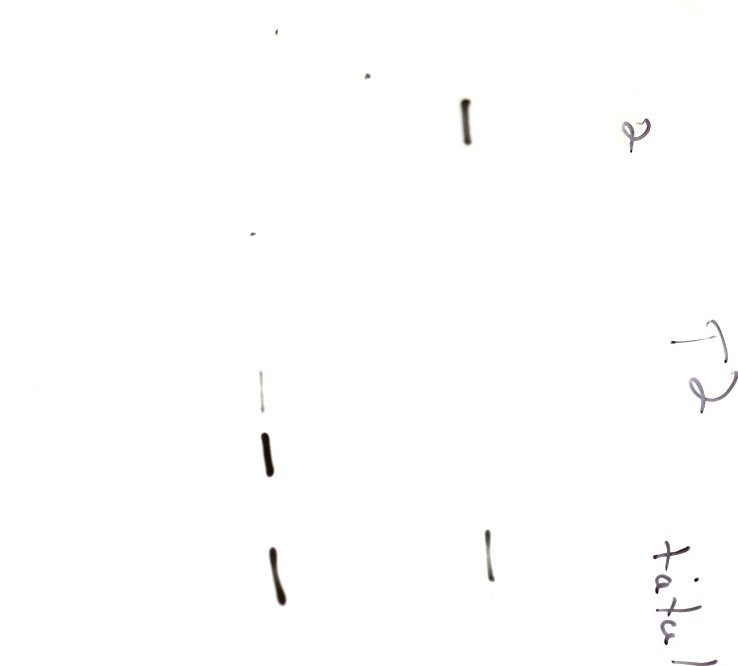

Supplement: Figure 1—figure supplement 2—source data 1. [file elife-75580-fig1-figsupp2-data1.zip › Figure 1-figure supplement 2 -source data 1.tif]

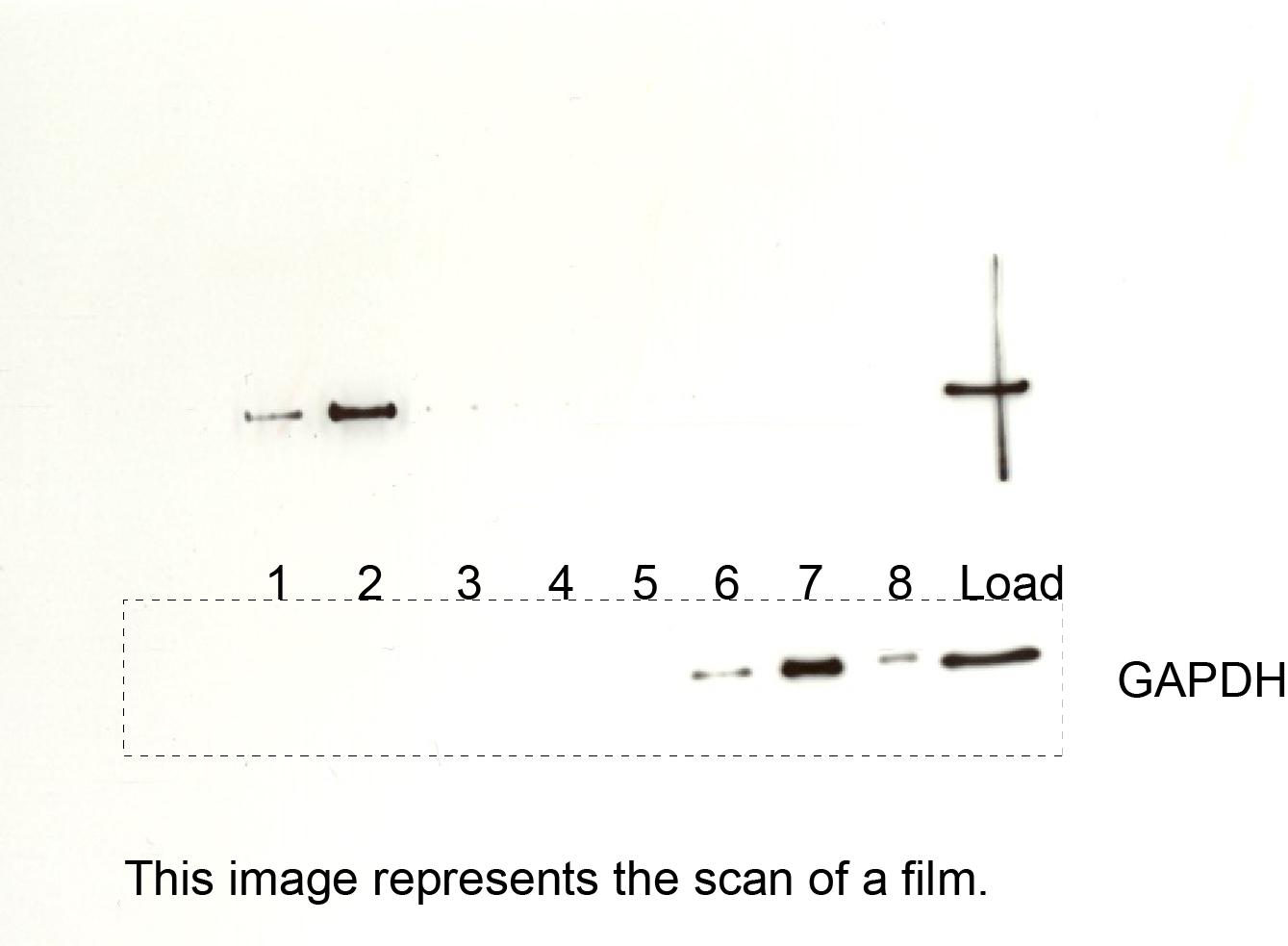

Supplement: Figure 1—figure supplement 2—source data 1. [file elife-75580-fig1-figsupp2-data1.zip › Figure 1-figure supplement 2 -source data 2.png]

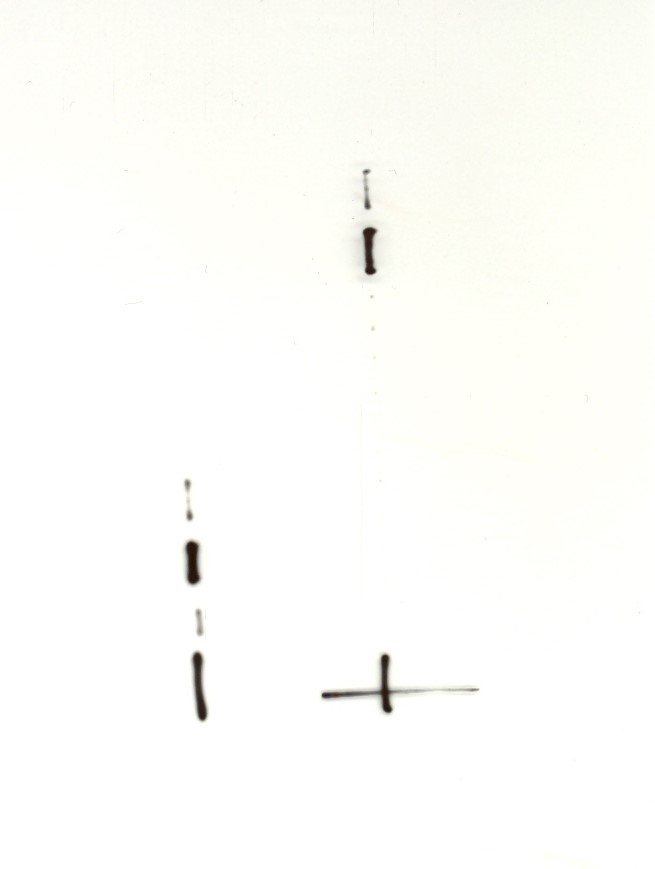

Supplement: Figure 1—figure supplement 2—source data 1. [file elife-75580-fig1-figsupp2-data1.zip › Figure 1-figure supplement 2 -source data 2.tif]

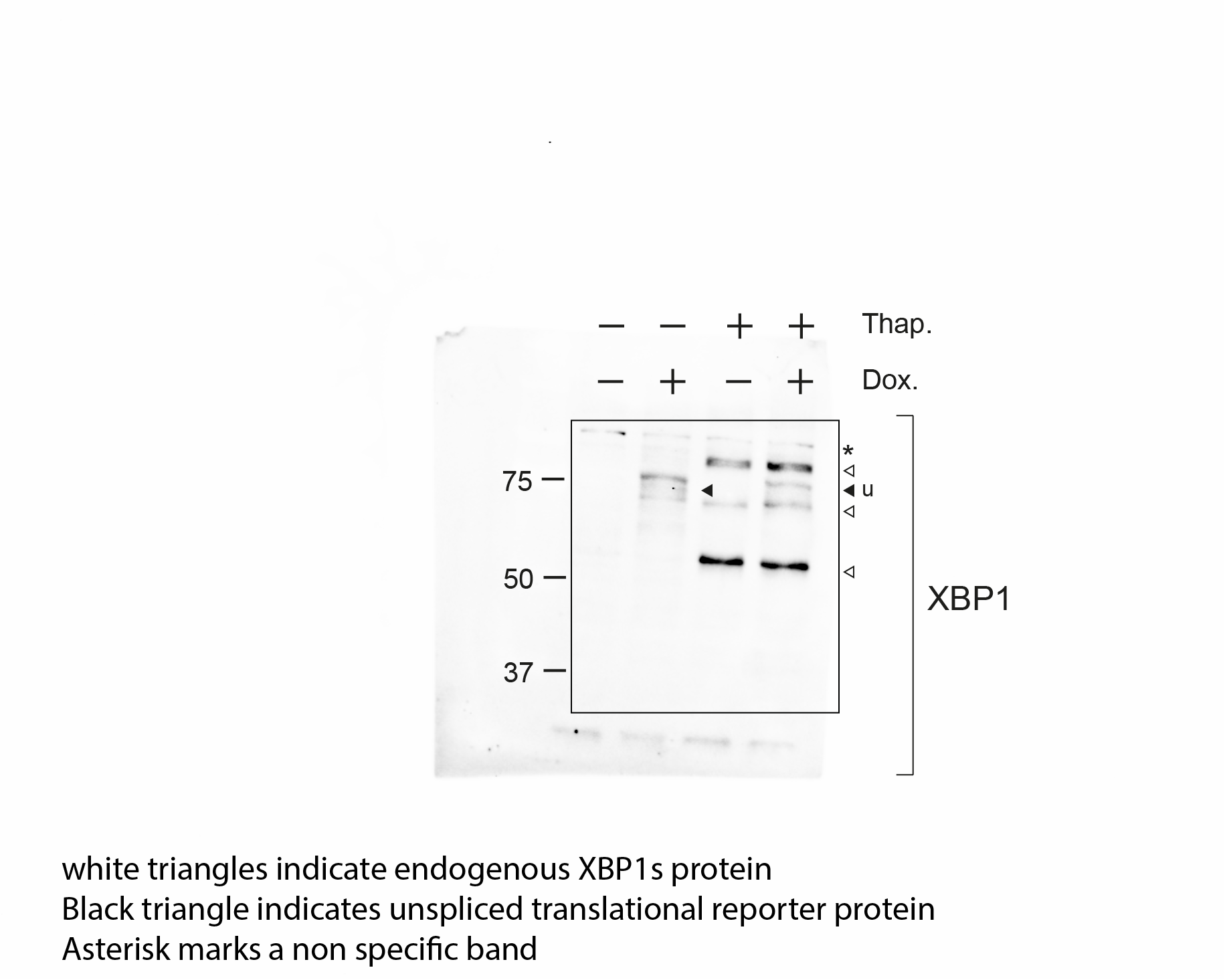

Supplement: Figure 2—source data 1. [file elife-75580-fig2-data1.zip › Figure 2-source data 1.png]

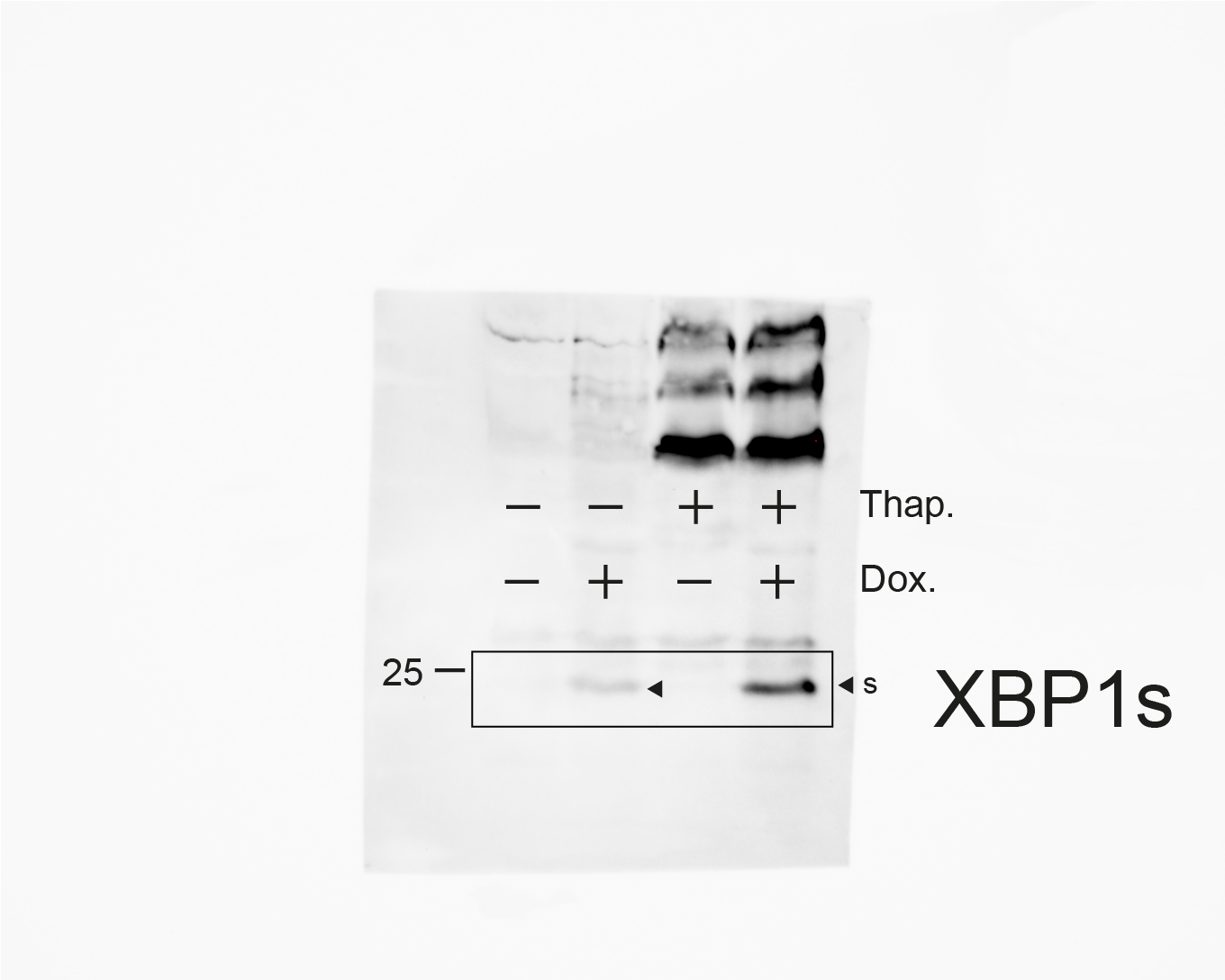

Supplement: Figure 2—source data 1. [file elife-75580-fig2-data1.zip › Figure 2-source data 2.png]

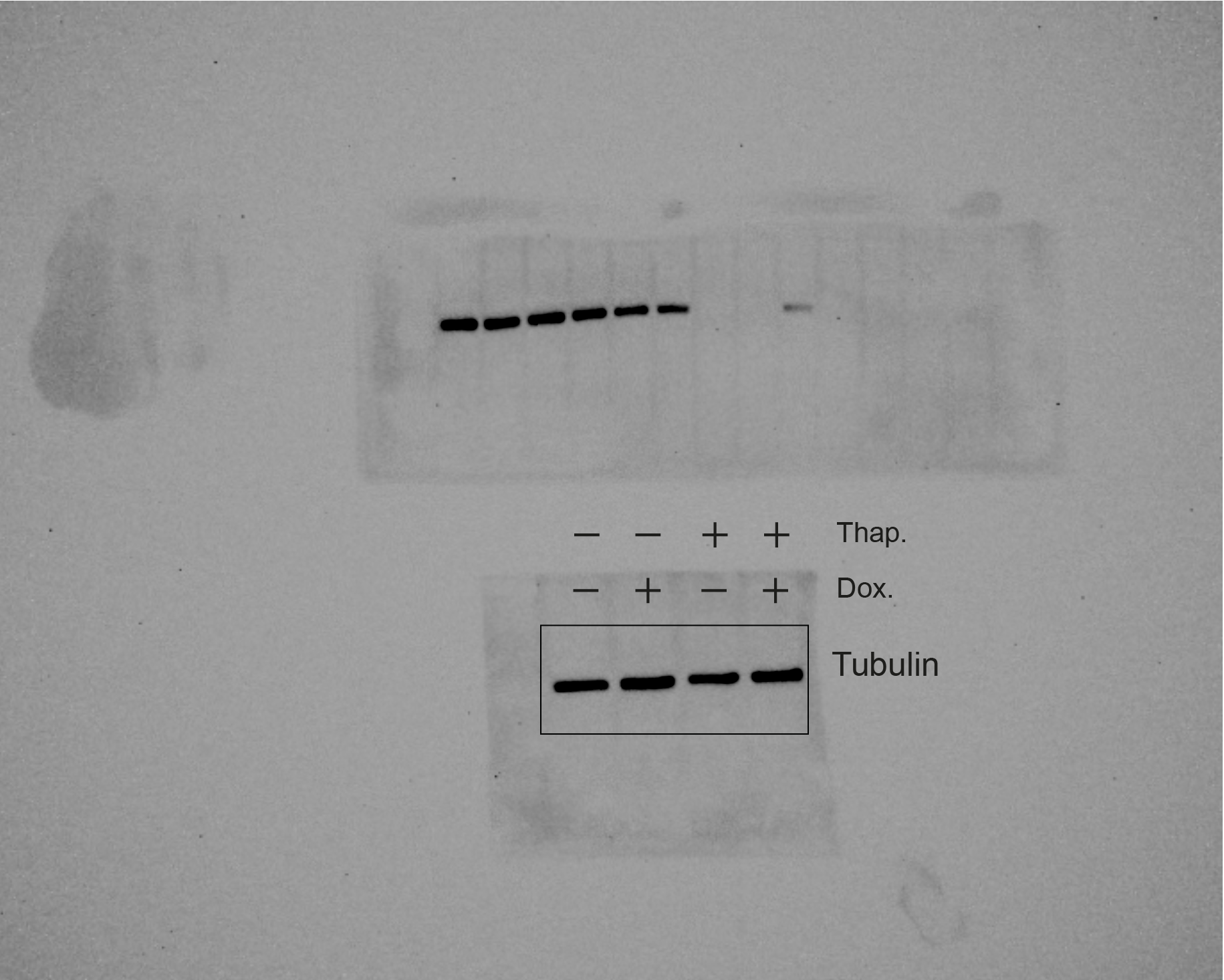

Supplement: Figure 2—source data 1. [file elife-75580-fig2-data1.zip › Figure 2-source data 3.png]

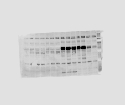

Supplement: Figure 3—figure supplement 1—source data 1. [file elife-75580-fig3-figsupp1-data1.zip › Figure 3-figure supplement 1-source data 1/0007979_01_TH.jpg]

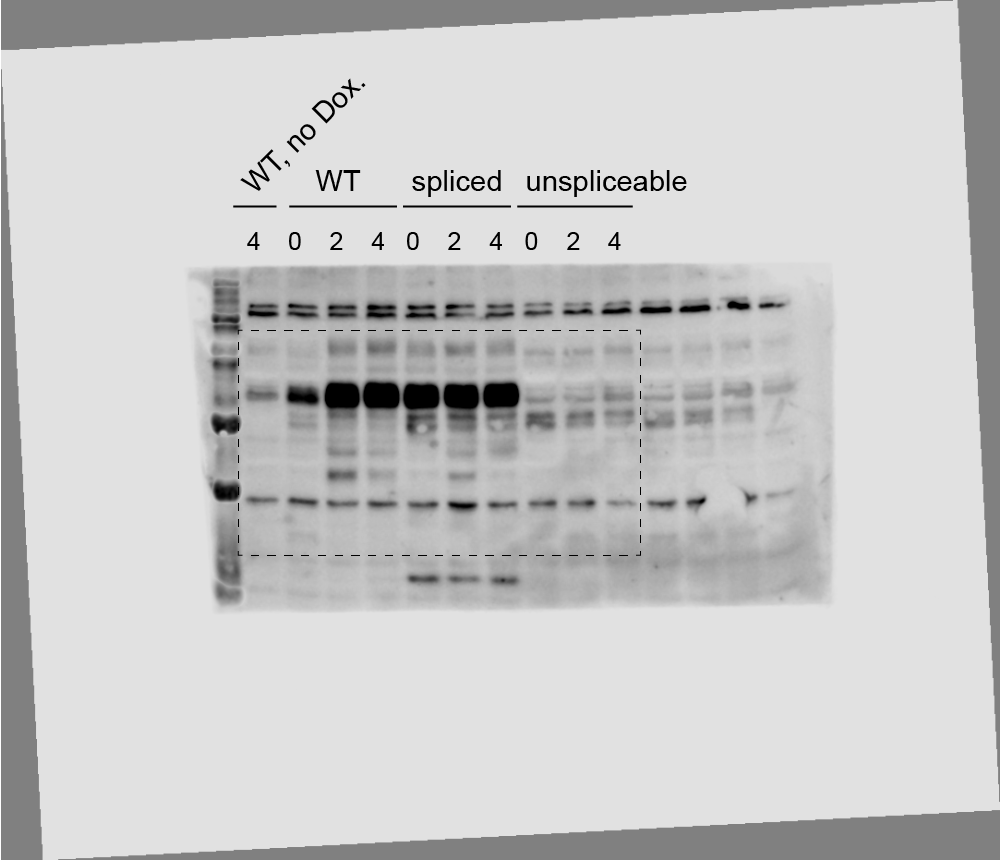

Supplement: Figure 3—figure supplement 1—source data 1. [file elife-75580-fig3-figsupp1-data1.zip › Figure 3-figure supplement 1-source data 1.png]

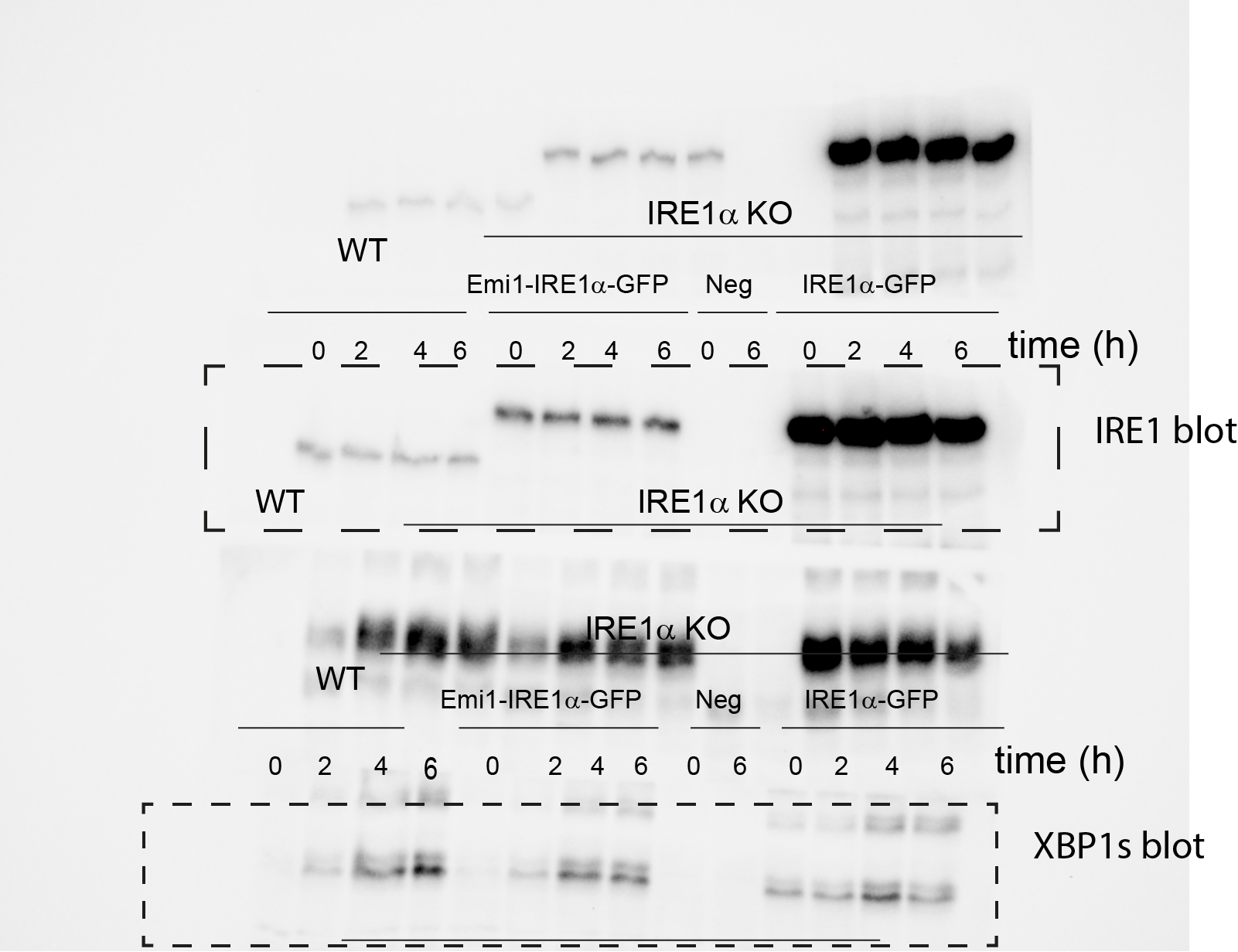

Supplement: Figure 4—source data 1. [file elife-75580-fig4-data1.zip › Figure 4_source data 1.png]

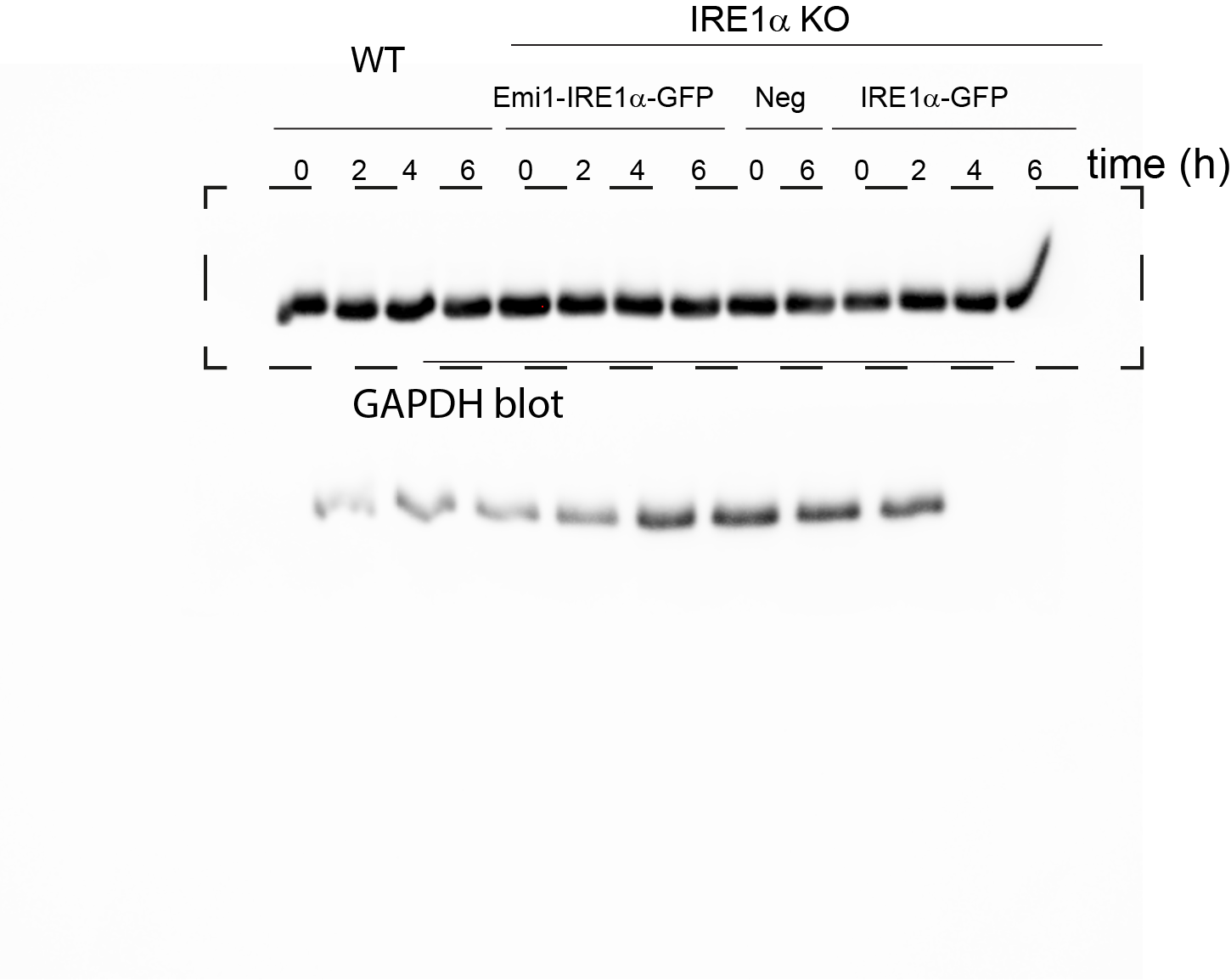

Supplement: Figure 4—source data 1. [file elife-75580-fig4-data1.zip › Figure 4_source data 2.png]

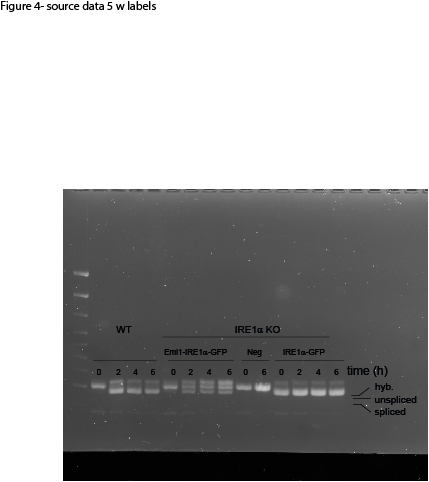

Supplement: Figure 4—source data 1. [file elife-75580-fig4-data1.zip › Figure 4_source data 3.jpg]

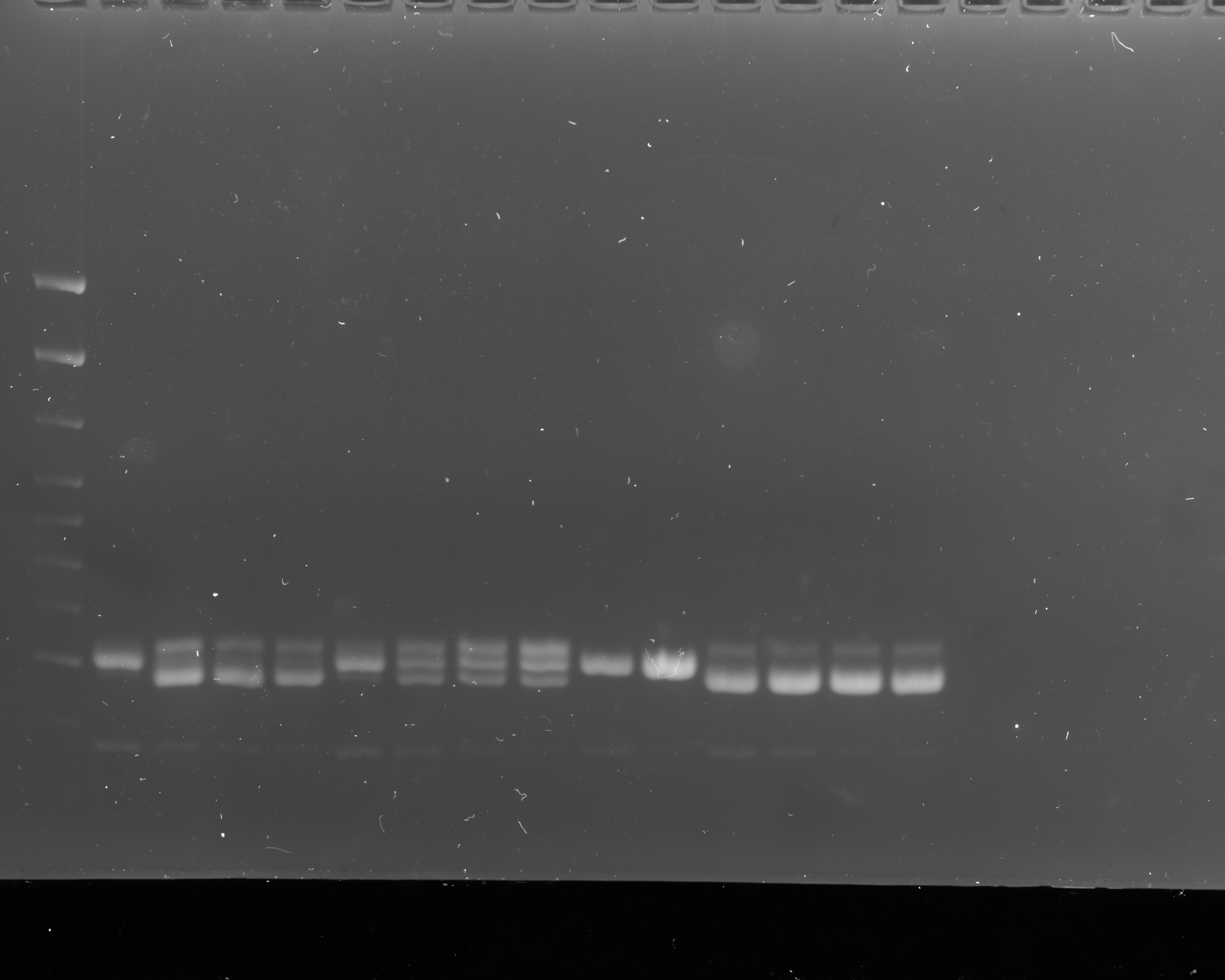

Supplement: Figure 4—source data 1. [file elife-75580-fig4-data1.zip › Figure 4_source data 3.tif]

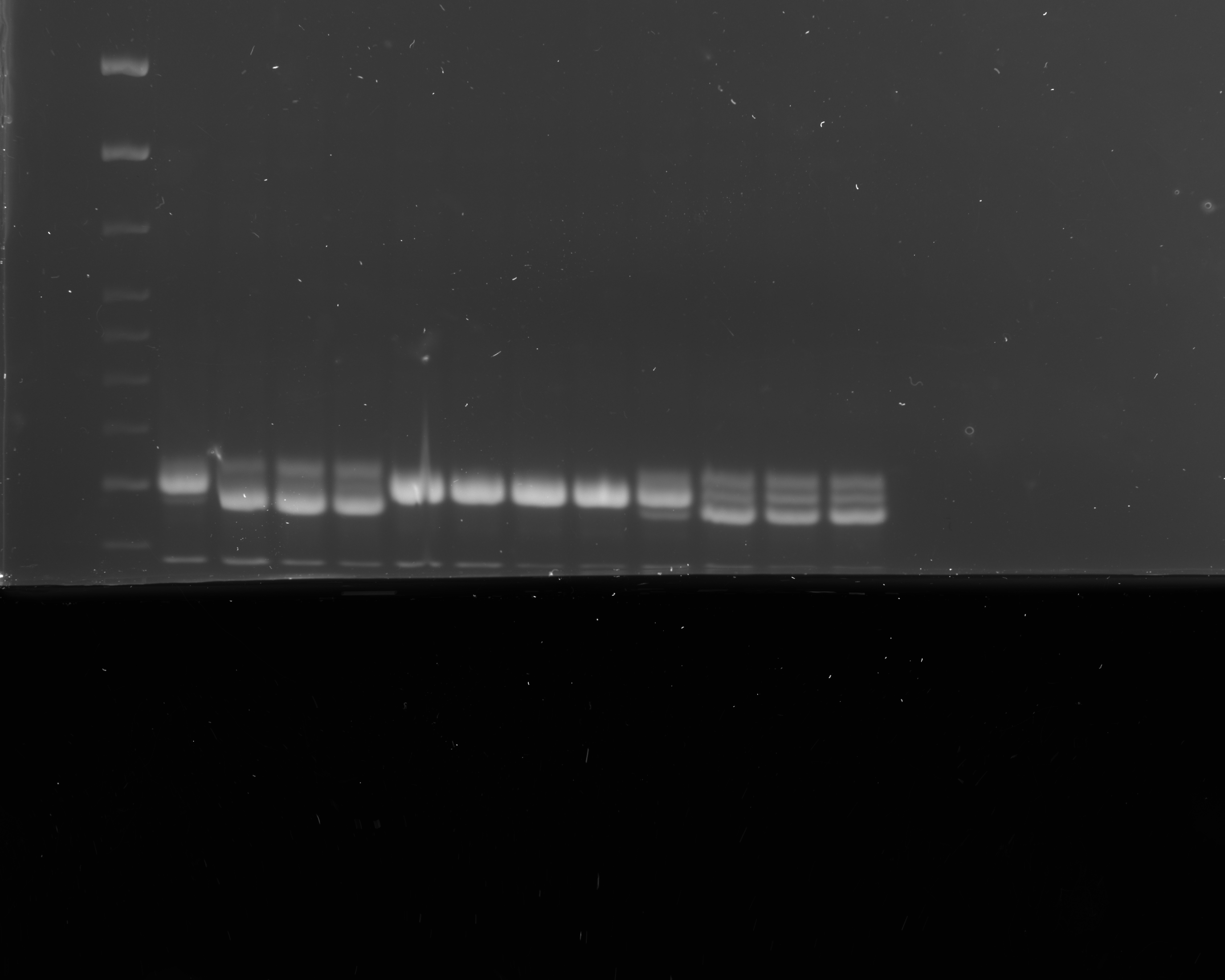

Supplement: Figure 4—figure supplement 1—source data 1. [file elife-75580-fig4-figsupp1-data1.zip › Figure 4 -figure supplement 1- source data 6.tif]

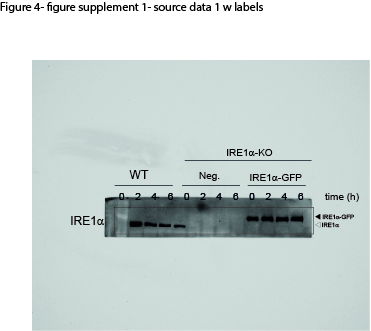

Supplement: Figure 4—figure supplement 1—source data 1. [file elife-75580-fig4-figsupp1-data1.zip › Figure 4- figure supplement 1- source data 1 w labels.jpg]

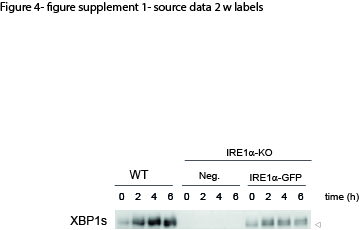

Supplement: Figure 4—figure supplement 1—source data 1. [file elife-75580-fig4-figsupp1-data1.zip › Figure 4- figure supplement 1- source data 2 w labels.jpg]

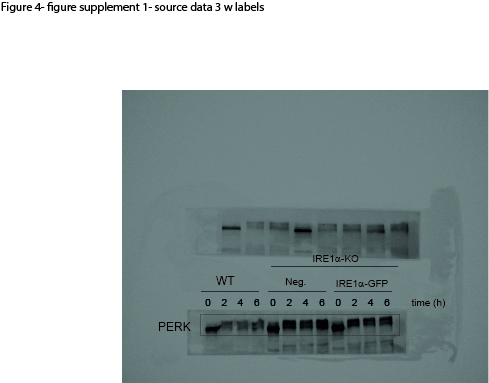

Supplement: Figure 4—figure supplement 1—source data 1. [file elife-75580-fig4-figsupp1-data1.zip › Figure 4- figure supplement 1- source data 3 w labels.jpg]

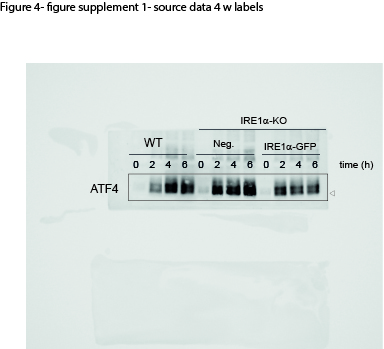

Supplement: Figure 4—figure supplement 1—source data 1. [file elife-75580-fig4-figsupp1-data1.zip › Figure 4- figure supplement 1- source data 4 w labels.jpg]

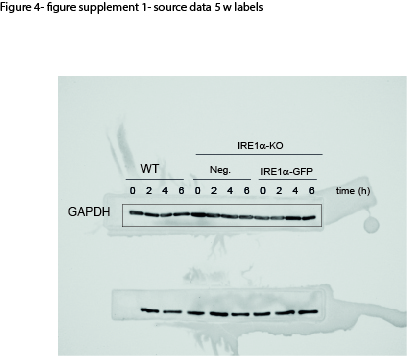

Supplement: Figure 4—figure supplement 1—source data 1. [file elife-75580-fig4-figsupp1-data1.zip › Figure 4- figure supplement 1- source data 5 w labels.jpg]

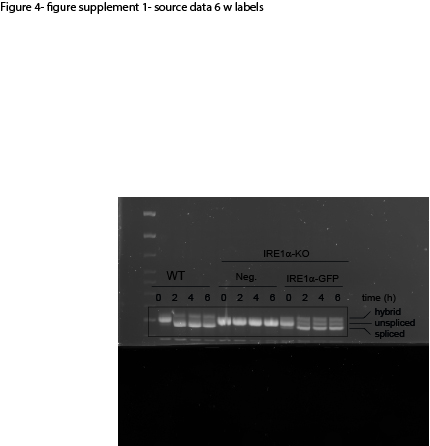

Supplement: Figure 4—figure supplement 1—source data 1. [file elife-75580-fig4-figsupp1-data1.zip › Figure 4- figure supplement 1- source data 6 w labels.jpg]
